# Seizures Are Regulated by Ubiquitin-specific Peptidase 9 X-linked (USP9X), a De-Ubiquitinase

**DOI:** 10.1371/journal.pgen.1005022

**Published:** 2015-03-12

**Authors:** Lily Paemka, Vinit B. Mahajan, Salleh N. Ehaideb, Jessica M. Skeie, Men Chee Tan, Shu Wu, Allison J. Cox, Levi P. Sowers, Jozef Gecz, Lachlan Jolly, Polly J. Ferguson, Benjamin Darbro, Amy Schneider, Ingrid E. Scheffer, Gemma L. Carvill, Heather C. Mefford, Hatem El-Shanti, Stephen A. Wood, J. Robert Manak, Alexander G. Bassuk

**Affiliations:** 1 Department of Pediatrics, The University of Iowa, Iowa City, Iowa, United States of America; 2 Interdisciplinary Program in Genetics, The University of Iowa, Iowa City, Iowa, United States of America; 3 Department of Ophthalmology and Visual Sciences, The University of Iowa, Iowa City, Iowa, United States of America; 4 Roy J. and Lucille A. Carver College of Medicine, The University of Iowa, Iowa City, Iowa, United States of America; 5 Department of Biology, The University of Iowa, Iowa City, Iowa, United States of America; 6 King Abdullah International Medical Research Center, King Abdulaziz Medical City, Riyadh, KSA; 7 Eskitis Institute for Drug Discovery, Griffith University, Brisbane, Australia; 8 Interdisciplinary Graduate Program of Neuroscience, Department of Veterans Affairs Medical Center, Iowa City, Iowa, United States of America; 9 School of Paediatrics and Reproductive Health, Robinson Institute, University of Adelaide, Adelaide, South Australia, Australia; 10 Epilepsy Research Centre, Department of Medicine, University of Melbourne, The Florey, Austin Health, Heidelberg, Melbourne, Australia; 11 Division of Genetic Medicine, Department of Pediatrics, University of Washington, Seattle, Washington, United States of America; 12 Qatar Biomedical Research Institute Medical Genetics Center, Doha, Qatar; 13 Interdisciplinary Graduate Program in Molecular and Cellular Biology, The University of Iowa, Iowa City, Iowa, United States of America; 14 Interdisciplinary Graduate Program in Neuroscience, The University of Iowa, Iowa City, Iowa, United States of America; The Jackson Laboratory, UNITED STATES

## Abstract

Epilepsy is a common disabling disease with complex, multifactorial genetic and environmental etiology. The small fraction of epilepsies subject to Mendelian inheritance offers key insight into epilepsy disease mechanisms; and pathologies brought on by mutations in a single gene can point the way to generalizable therapeutic strategies. Mutations in the PRICKLE genes can cause seizures in humans, zebrafish, mice, and flies, suggesting the seizure-suppression pathway is evolutionarily conserved. This pathway has never been targeted for novel anti-seizure treatments. Here, the mammalian PRICKLE-interactome was defined, identifying prickle-interacting proteins that localize to synapses and a novel interacting partner, USP9X, a substrate-specific de-ubiquitinase. PRICKLE and USP9X interact through their carboxy-termini; and USP9X de-ubiquitinates PRICKLE, protecting it from proteasomal degradation. In forebrain neurons of mice, USP9X deficiency reduced levels of Prickle2 protein. Genetic analysis suggests the same pathway regulates Prickle-mediated seizures. The seizure phenotype was suppressed in *prickle* mutant flies by the small-molecule USP9X inhibitor, Degrasyn/WP1130, or by reducing the dose of *fat facets* a *USP9X* orthologue. *USP9X* mutations were identified by resequencing a cohort of patients with epileptic encephalopathy, one patient harbored a *de novo* missense mutation and another a novel coding mutation. Both *USP9X* variants were outside the PRICKLE-interacting domain. These findings demonstrate that USP9X inhibition can suppress *prickle*-mediated seizure activity, and that *USP9X* variants may predispose to seizures. These studies point to a new target for anti-seizure therapy and illustrate the translational power of studying diseases in species across the evolutionary spectrum.

## Author Summary

Epilepsy is a common disabling disorder characterized by seizures with complex genetic and environmental components. The absence of a definitive pathophysiology for epilepsy stymies the development of effective treatment strategies. In a small fraction of epilepsy cases however, single gene mutations may illuminate seizure-causing mechanisms, which may open the door to the discovery of broader, more effective therapeutic strategies. We have previously shown that disruption of *Prickle* genes in multiple species including humans, results in a predisposition to seizures. Those findings support Prickle in a seizure-preventing role and presents a possible anti-seizure therapeutic target. We identified the deubiquitinase Usp9x (ubiquitin-specific peptidase 9 X-linked) as a new Prickle binding partner which stabilized Prickle by preventing its degradation. In mice lacking the Usp9x protein in their forebrains, Prickle2 was barely detectable. In seizure-prone *prickle* mutant *Drosophila*, reducing *fat facets* (*Drosophila* usp9x) genetically or by a small-molecule usp9x inhibitor (Degrasyn/WP1130) suppressed the seizures. We also found 2 epilepsy patients harboring mutations in *USP9X*. Our findings demonstrate that inhibition of Usp9x can arrest *prickle*-mediated seizures, and variations in *USP9X* may predispose to seizures. From these studies, we have elucidated a seizure-inducing mechanism, identified a potential anti-seizure target, and a potential anti-seizure drug.

## Introduction

Mutations in the PRICKLE genes can cause seizures in humans, zebrafish, mice, and flies, suggesting the seizure-suppression pathway is evolutionarily conserved.[1–5]. Prickle binding partners have been studied extensively only in either non-neuronal vertebrate cell lines or non-neuronal tissues in the fly. (In both cases Prickles were shown to bind other WNT/PCP proteins.[6, 7]) Such targeted approaches showed Prickles interact with REST, some kinases (including BCR), and post-synaptic density proteins, including TANC1 and TANC2.[4, 6] To identify neuronal proteins that bind Prickles, recent work by our group and others showed PRICKLE1 also binds to Smurf1 (a ubiquitin ligase),[8] and Synapsin1, (a gene implicated in both epilepsy and autism)[1]; and PRICKLE2 also binds PSD-95 and p150^Glued^.[9] To identify other PRICKLE binding partners in neuronal-like cells, we used mass spectroscopy: a more global, unbiased approach.

## Results

**Fig 1:**
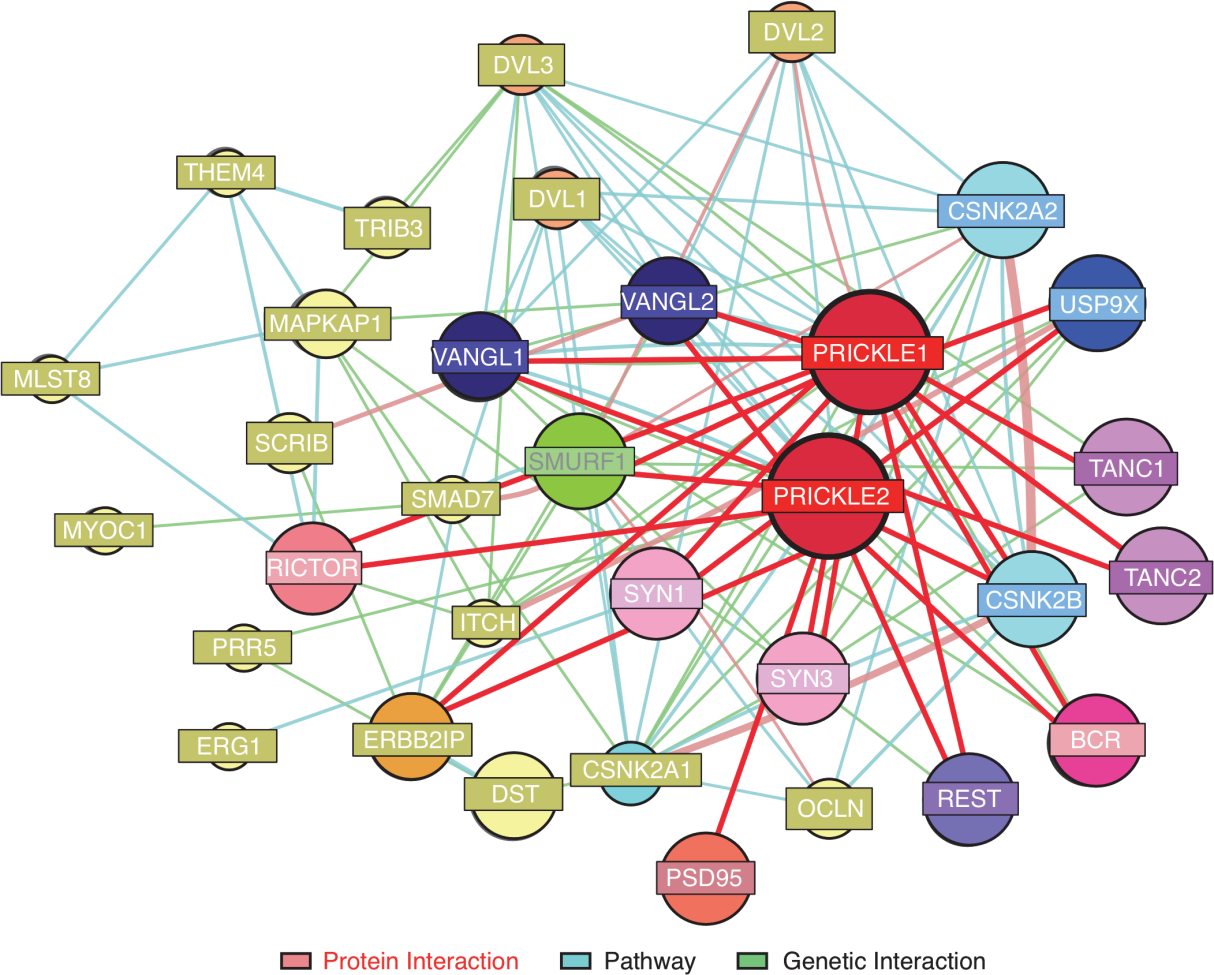
Prickle interactome. We combined findings from our proteomics interaction experiment and public databases to generate a prickle interactome. We used the MetaCore (MetaCore, GeneGO Inc., St. Joseph, MI, USA) networking function and String database 9.1 to curate interaction maps of the proteins identified. Information for identified interactions is obtained from several sources including but not limited to genomic context, database imports (PPI and pathway databases), high-throughput experiments, co-expression, and text mining. We uploaded our lists of proteins from LC-MS/MS into the software programs and exported the networks into Cytoscape 2.7.0 for manipulation of the network appearance. (Nodes, circles; Edges, lines). Red lines correspond to interactions observed by our labs using yeast-2-hybrid and IP-MS approaches. The extended interactome was generated as we have previously described. [1, 6–9, 18, 19] Prickle1 and Prickle2 interact with known synaptic proteins. The interaction with USP9X is novel.

The interaction was monitored in a subclone of rat pheochromocytoma PC12 cells which, when treated with Nerve Growth Factor (NGF), assume a sympathetic neuron-like phenotype.[10] Clonal PC12 lines that overexpressed doxycycline-inducible GFP, GFP-PRICKLE1 (GFP-PK1), or GFP-PRICKLE2 (GFP-PK2) were produced (S1 Fig). Protein complexes immunoprecipitated with anti-GFP beads from whole-cell lysates (S1C Fig) were analyzed by mass spectrometry (IP-MS, S2 Fig).[1] Prickle interactors were considered candidates only if they recovered >10 peptide matches with both GFP-PRICKLE1 and GFP-PRICKLE2, but not with GFP alone (S1 Table). This dataset recovered both known Prickle-interactors (e.g., Tanc2, and Bcr[6]) and novel Prickle interactors, including Usp9x,[11–15] a substrate-specific de-ubiquitinase. The putative Prickle-Usp9x interaction was of particular interest because Usp9x physically interacts with Smurf1 (to date, one of the few Prickle interactors identified in neural tissues[13] and both are implicated in neurite extension).[16] Moreover, since ubiquitination plays a role in cancer pathogenesis, a variety of reagents that modulate this system are already commercially available and in clinical trials.[17] The combination of previously identified Prickle-interacting partners with the present studies are depicted in the Prickle-interactome (Fig. 1) that we utilize to identify new seizure-modifying targets.

**Fig 2:**
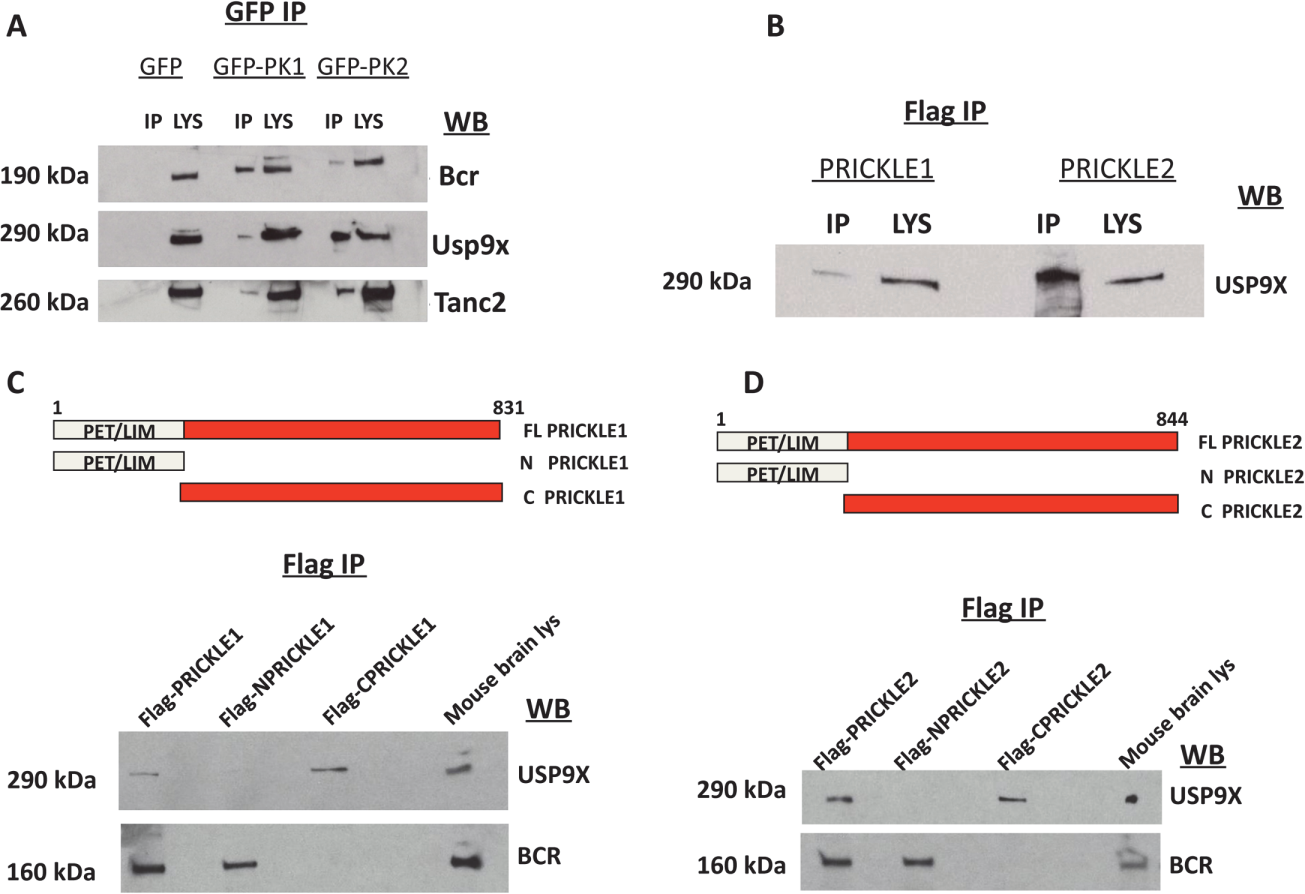
PRICKLE interacts with USP9X via its carboxyl terminal. **A.** PRICKLE1 and PRICKLE2 interact with endogenous Bcr, Tanc2, and Usp9x in NGF-differentiated PC12 cells. GFP immunoprecipitates from stable lines expressing GFP, GFP-PRICKLE1 or GFP-PRICKLE2 confirm that PRICKLE interacts with Bcr, Usp9x and Tanc2. **B**. Flag immunoprecipitates, from HEK293T cells overexpressing flag-tagged PRICKLE1 or PRICKLE2, show endogenous USP9X physically interacts with PRICKLE. **C, D.** Schematic of PRICKLE1(C) and PRICKLE2(D) constructs. Flag-immunoprecipitates from HEK293T cells overexpressing the indicated constructs were analyzed by anti-USP9X Western blotting. Both PRICKLE 1 and 2 interact with USP9X via their C-termini while BCR binding mapped to their N-termini.

To evaluate further USP9X as a PRICKLE binding partner, coimmunoprecipitation (co-IP) assays were carried out in the original PC12 GFP, GFP-PRICKLE1, and GFP-PRICKLE2 cells lines, utilizing Tanc2 and Bcr as positive controls. To detect the respective endogenous proteins, GFP-immunoprecipitates from the differentiated cell lines were immunoblotted with anti-BCR, anti-TANC2, and anti-USP9X. Fig. 2A demonstrates interaction by all three proteins in GFP-PRICKLE1 and GFP-PRICKLE2 immunoprecipitates. To validate this interaction, co-IPs were carried out in a different cell line. Flag-tagged PRICKLE1 and PRICKLE2 were immunoprecipitated from human embryonic kidney (HEK293T) cells and subjected to anti-USP9X Western blot analysis; Fig. 2B shows both PRICKLE1 and PRICKLE2 interact with endogenous USP9X in HEK293T cells.

To map the PRICKLE1-USP9X interacting domain, recombinant vectors expressing full-length, N-terminal or C-terminal portions of PRICKLE1 (schematic diagrams in Fig. 2C, D) were transfected into HEK293T cells and immunoprecipitated via the Flag tag. Fig. 2C showed that endogenous USP9X interacted specifically with the PRICKLE1 C-terminus. On the other hand, BCR interacted with the N-terminus, a region that includes the PET/LIM domains, previously known to mediate Prickle1 protein-protein interactions.[20] The same approach defined the PRICKLE2-USP9X interacting domain. As with PRICKLE1, while the interaction with BCR mapped to the N-terminus, the PRICKLE2–USP9X interaction mapped to the C-terminus of PRICKLE2 (Fig. 2D).

**Fig 3:**
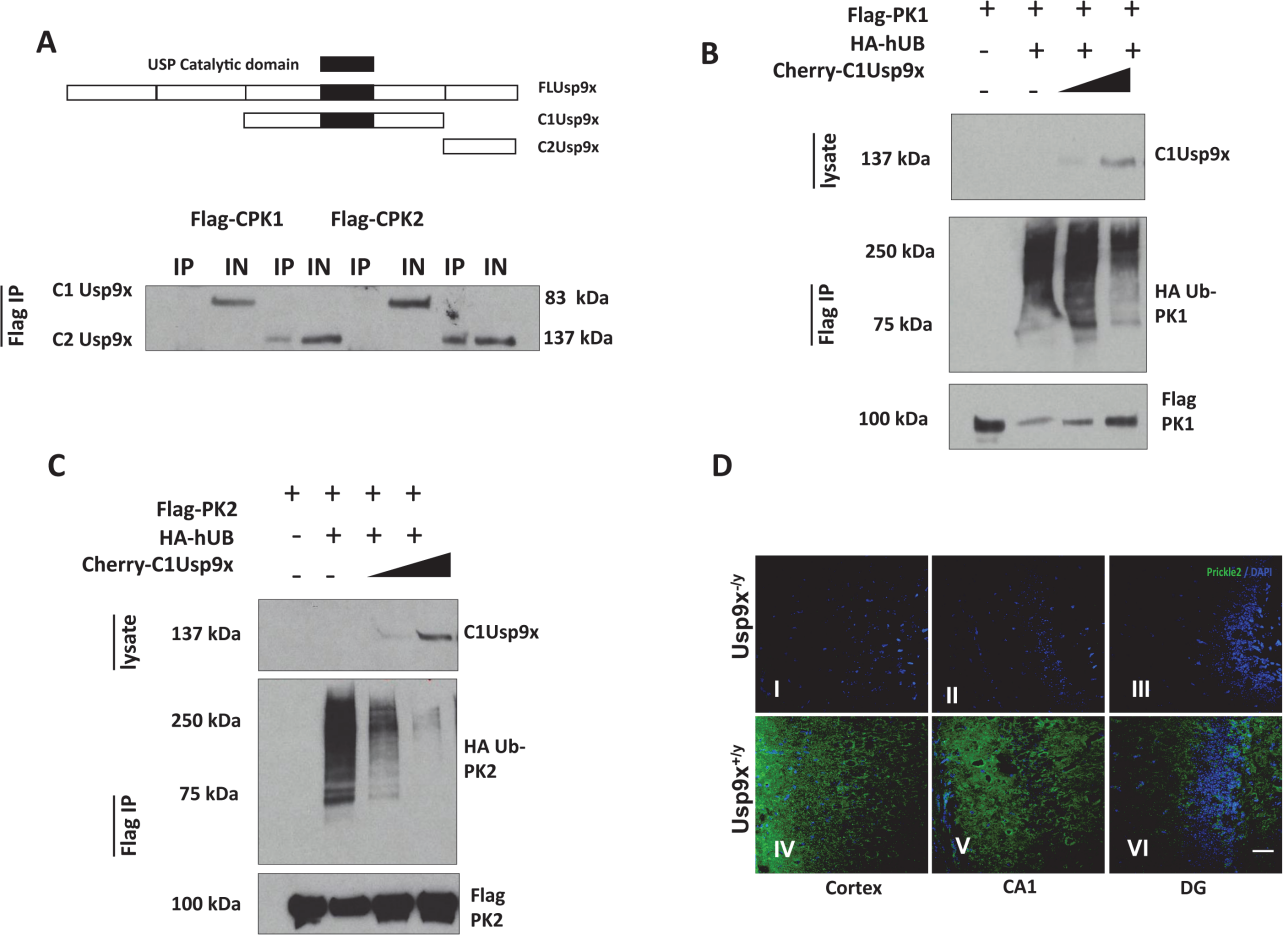
USP9X stabilizes PRICKLE in HEK293T cells and the mouse brain. **A**. Flag-immunoprecipitates from HEK293T cells overexpressing the indicated constructs show PRICKLE and USP9X interact via their carboxyl termini. **B, C.** USP9X deubiquitinates PRICKLE. Immunoprecipitates from HEK293T cells overexpressing the indicated constructs show PRICKLE ubiquitination in the presence of HA-Ubiquitin and deubiquitination/stabilization by Usp9x. IP (immunoprecipitates), IN (input). **D.** Loss of Usp9X affects Prickle2 (green) expression. Deletion of Usp9x results in decreased Prickle2 expression in the cortex (I), CA1 (II) and dentate gyrus (III) of 4-week old mice when compared to controls (IV, V, VI). n = 2. Scale bar:20μM. Nuclear stain = DAPI.

SMURF1 and USP9X are known to physically interact via the second WW domain of SMURF1 and the USP9X carboxyl terminus (a fragment named C2)[13] so, hypothetically, the PRICKLE interaction should also map to the same region. To test this, a PRICKLE1 or PRICKLE2 c-terminal fragment was expressed with one of two flag-tagged Usp9x deletion fragments: C1Usp9x (amino acids 1216–2107) and C2Usp9x (the carboxy terminal amino acids 2102–2560; Fig. 3A). Complexes were immunoprecipitated via the Flag tag. Fig. 3A shows that the PRICKLE1 and PRICKLE2 interactions also map to the carboxyl terminus of Usp9x (C2). The C-terminal end of both PRICKLEs and USP9X are therefore crucial for the PRICKLE-USP9X interaction.

PRICKLE is ubiquitinated by the E3 ligase, SMURF2, which promotes PRICKLE degradation and turnover in HEK293T cells.[8] Ubiquitin tags from mono- and polyubiquitinated proteins can be removed by the C1-terminal catalytic motif of USP9X deubiquitinase.[12, 13, 21–23] We postulated the physical interaction between the PRICKLEs and USP9X might indicate that PRICKLE was a USP9X substrate, and that USP9X deubiquitinates and stabilizes both PRICKLE1 and PRICKLE2. To test this idea, PRICKLE substrates were monitored in USP9X deubiquitination assays.

In previous studies, overexpression of the C1Usp9x catalytic fragment alone was sufficient to deubiquitinate USP9X substrates.[23] Accordingly, the robust ubiquitination of Flag-PRICKLE1 (Fig. 3B, C) was antagonized by overexpression of C1Usp9x; and PRICKLE was stabilized (Fig. 3). Similar results were obtained with full-length USP9X (S3 Fig). In the presence of the proteasomal inhibitor LLNL (N-acetyl-L-leucyl-L-leucyl-L-norleucinal), polyubiquitinated PRICKLE1 or PRICKLE2 accumulated, indicating that degradation of ubiquitinated PRICKLE is mediated by the proteasome as opposed to the lysosome (S3 Fig). To determine if a Prickle-Usp9x interaction has a functional effect in mice, we generated mice with deletion of Usp9x in the forebrain (Emx1-Cre/Usp9x loxtemp)[12] and examined expression of Prickle2. In the absence of Usp9x in 4-week old mice, Prickle2 was not detected in the cortex, CA1 region of the hippocampus, and dentate gyrus (Fig. 3D). Taken together, these data suggest that Prickles are novel Usp9x substrates.

**Fig 4:**
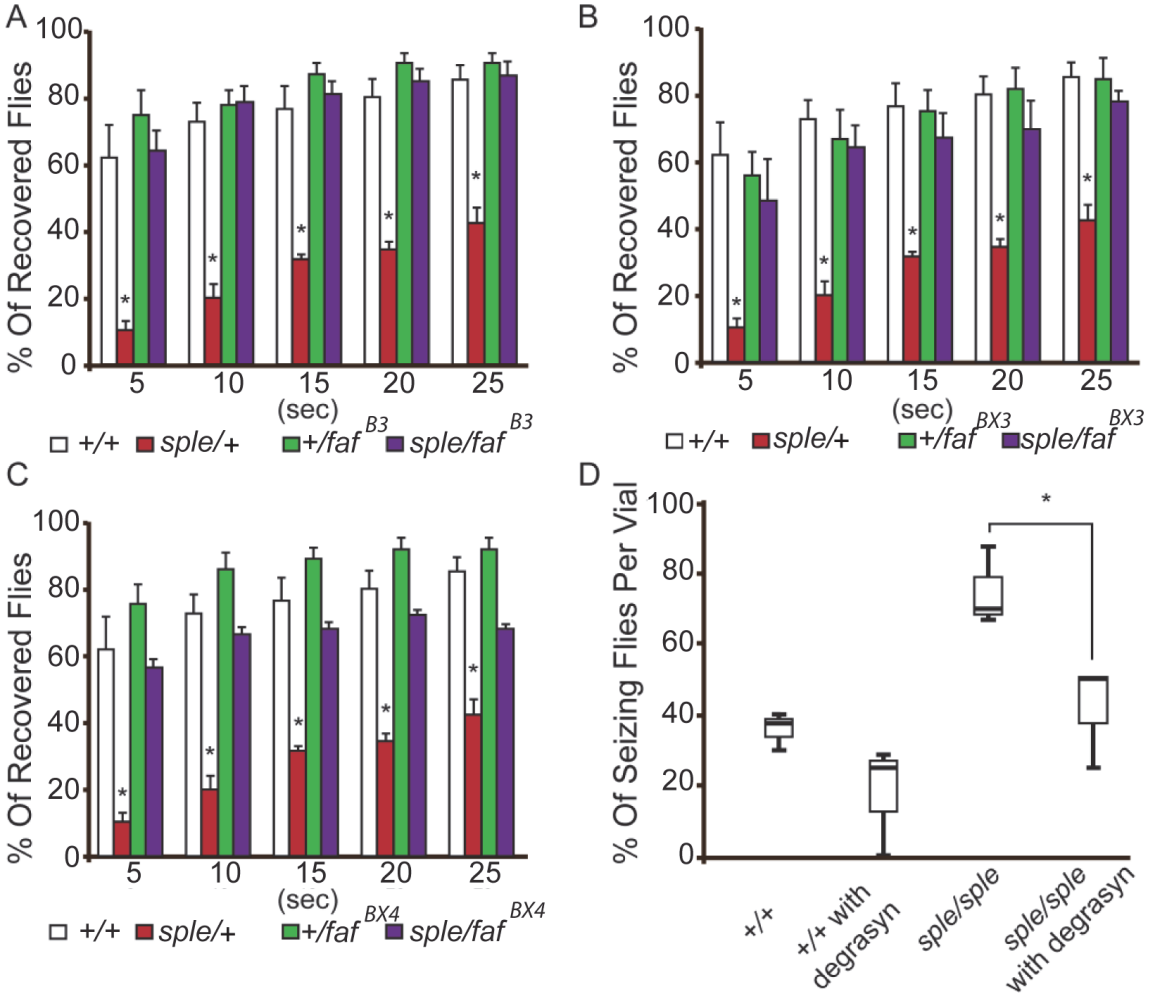
Genetic and pharmacological suppression of the seizure phenotype in prickle mutant flies. (**A–C**) Reducing faf dosage with three separate loss-of-function alleles ((faf^B3^, faf^BX3^, faf^BX4^; graphs A, B and C, respectively) suppresses the pk^sple^-mediated seizure phenotype detected by the modified bang sensitivity assay. Six vials per genotype were assayed. **D**. Inhibition of faf activity with Degrasyn suppresses pk^sple^-mediated seizure activity detected using the “fly flip” assay. Three vials per genotype were assayed. Note: +/+ = Oregon-R control flies; sple = pk^sple^; sple/faf = pk^sple^/+; +/faf. * = p < 0.05. Error bars = standard error. Each vial contains 5 males and 5 females.

Genetic analysis was used to determine if Prickle and Usp9x interact genetically in fruit flies. We reduced the dosage of the *USP9X* orthologue (*fat facets*, or *faf*) in the context of a *prickle* mutation (*pk* ^*sple*^/+) which on its own promotes both behavioral and electrophysiologic seizure activity.[3, 5] Three separate loss-of-function *faf alleles* were used to create transheterozygotes, which were then assayed with the bang sensitivity behavioral assay to assess recovery time from seizure activity. For every *faf* allele used, reducing its level suppressed the seizures in the context of the *pk* ^*sple*^/+ flies (Fig. 4A-C). Adding the USP9X small-molecule inhibitor Degrasyn/WP1130[24] to the fly food inhibited seizure activity in *pk* ^*sple*^ homozygous flies (Fig. 4D) using a “fly flip” assay (see Materials and Methods).

The demonstration that pharmacologic treatment with a USP9X inhibitor suppresses seizures in conjunction with genetic seizure suppression utilizing fly *USP9X* orthologue mutations suggests that *USP9X* mutations could protect humans from seizures. In contrast to this suggestion, in humans, *USP9X* has been postulated to be an epilepsy candidate gene. *USP9X* is X-linked; and in males has been associated with both autism spectrum disorder (ASD) and intellectual disability (which are frequently co-morbid with epilepsy). Moreover, mice with Usp9x-deficient neurons develop abnormal neuronal connectivity.[12, 14, 25, 26] A similar apparent discrepancy has been observed in the case of the *SCN1A* gene (which codes for the NAV1.1 channel) and its fly homologue *para*. Here, the great majority of 68 different mutations in *para* suppress seizures in the fly,[27–31] yet human *SCN1A* mutations are associated with severe epileptic encephalopathies, febrile seizure syndromes, and Dravet syndrome. Recent studies demonstrated that although loss-of-function *para* alleles suppress seizures, a few recently identified gain-of-function *para* alleles (e.g., *para* ^*bss1*^) actually cause seizures in the fly.[27] Accordingly, many of the most severe human *SCN1A* mutations are missense or truncation, gain-of-function alleles (see web resources).[32]

To assess if *USP9X* coding variants might be associated with human seizures, the *USP9X* gene was resequenced in 284 male patients with epileptic encephalopathy. One male patient, T17133, presented with epileptic encephalopathy and was found to carry a *de novo USP9X* mutation (c.3034T>C, p.Ser1012Pro) at a highly conserved residue (GERP 5.62) that was predicted to be possibly damaging by PolyPhen (0.898). Another patient with infantile spasms (parental genotypes unavailable) harbored variant (T2587c.5669G>A, p.Gly1890Glu) a rare missense variant. Both mutations lie outside the identified Prickle-binding domain. The USP9X pSer1012 is very close to the Ubiquitin-like (UBL) domain; and the USP9X Gly1890 is in UCH (Ubiquitincarboxyl-terminal hydrolase) domain, just eleven amino acids from the proton acceptor in the active site, and in between the catalytic Cys and His motifs that form the catalytic domain, suggesting that both mutations likely alter normal USP9X function. Rare coding variants were sought in the cohort of 284 males with epilepsy and the male exomes in the NHLBI exome variant server EVS (see web resources) in a one-tailed Chi-squared analysis. For the combined African American and European American cohorts, we found a statistical association (p = 0.0350) between *USP9X* rare coding variants and epilepsy (S2 Table). This further supports the assertion that *USP9X* may be a novel epilepsy gene.

## Discussion

Our studies suggest USP9X-mediated de-ubiquitination stabilizes PRICKLE proteins in the nervous system. These results are consistent with results in other tissues, where *faf* RNAi reduced Prickle levels in *Drosophila* apicolateral junctions,[33] as well as studies showing USP9X stabilizes several proteins involved in various aspects of neuronal development and cancer.[12] Notably, USP9X was shown to stabilize oncoproteins (e.g., MCL-1 and p53), sparking interest in modulating USP9X in tumors, with promising results.[24]

Here, we showed that human *USP9X* mutations are associated with epilepsy. Our data corroborate other evidence showing *USP9X* mutations in males with co-morbid epilepsy conditions (e.g., intellectual disability and ASD). In addition to USP9X, several other members of our identified Prickle-interactome were already implicated in seizures, intellectual disability, and ASD. (For example, a *TANC2* variant in a patient with intellectual disability and febrile seizures[34] and in a patient with ASD,[35] and a *SMURF1* variant in a patient with epileptic encephalopathy).[36]

*PRICKLE* mutant humans, mice, zebrafish, and *Drosophila* all exhibit seizures.[3, 5] In flies, the *prickle* seizure phenotype can be genetically suppressed by reducing Faf (creating transheterozygotes) or pharmacologically rescued by treating *pk* ^*sple*^ mutants with Degrasyn/WP1130. Although USP9X is genetically associated with seizures in both flies and humans, mutations are seizure-protective in the flies but seizure-inducing in humans. This opposing effect is likely because the identified human *USP9X* mutations are all protein-changing mutations that reside outside of the Prickle-interaction domain whereas the fly *faf* mutations that suppress seizures are all amorphic alleles. This suggests that, for *USP9X*, complete loss of function may have a different phenotype than protein coding alleles. Before our study, neither USP9X inhibition nor stimulation was identified as a potential anti-seizure pathway. Yet, given the evolutionarily conservation of the Prickle-pathway, our results suggest Degrasyn/WP1130 and other USP9X-modulating molecules should be pursued as novel therapeutic agents for patients suffering from seizures.

## Materials and Methods

### Antibodies and antibody-conjugated agarose beads

The antibodies employed were rabbit polyclonal anti-BCR (Santa Cruz), rabbit polyclonal anti-TANC2 (Bethyl), polyclonal rabbit-anti USP9X (Abcam), mouse monoclonal anti-β-actin (Sigma), mouse monoclonal anti-Flag (Sigma), rat monoclonal anti-HA-peroxidase (Roche), rabbit polyclonal anti-dsRed from Clontech (detects mCherry) mouse monoclonal anti-Myc (Santa Cruz), rabbit polyclonal anti-GFP (Santa Cruz). Anti-rabbit and anti-mouse horseradish peroxidase (HRP)-conjugated secondary antibodies (Thermo Scientific). The agarose antibody-conjugated beads were protein A/G beads (Pierce), anti-Flag (Santa Cruz), and anti-GFP

### Plasmids

Flag-PRICKLE1 plasmid was as previously described.[4] The N-terminus of PRICKLE1 (aa 1 to 313) and the C-terminus of PRICKLE1 (aa 314 to 831) were each Flag-tagged on the C-terminus and cloned into EcoRI (5’) and KpnI (3’) restriction sites of the pcDNA3.1 vector. The N-terminus (aa 1 to 317) and C-terminus of PRICKLE2 (aa 318 to 844) were also Flag-tagged on the C-termini and cloned into EcoRI (5’) and KpnI (3’) restriction sites of the same vector.

Using previous studies as a guide, we cloned deletion constructs of murine Usp9x.[23] C1Usp9x aa 1216–2107 (with the catalytic domain) and C2Usp9x aa 2102–2560 (the carboxyl terminus) were cloned into the BamH1 and BSpe1 restriction sites of the pmCherryC1 plasmid (Clontech). The previously described HA-UbiquitinC plasmids were kindly gifted by Pedro Gonzalez-Alegre (University of Iowa, Iowa City).

### Tissue culture

HEK293T[37] cells were cultured in DMEM (Dulbecco’s modified eagle medium; Gibco) supplemented with 10% FBS (fetal bovine serum) and 1% penicillin-streptomycin (Gibco). PC12 cells were cultured in collagen-coated plates in RPMI (Roswell Park Memorial Institute medium) supplemented with 5% FBS, 10% HS (horse serum), 1% penicillin-streptomycin, hygromycin (100μg/ml), and blasticidin (5μg/ml). Cells were maintained in a humidified 37°C, 5% CO_2_ incubator. Transgene expression and differentiation were induced by 1.5μg/ml dox and 100 ng/ml NGF treatment in low serum RPMI medium (2% HS, 1% FBS) respectively.

### Generation of PC12 inducible lines expressing GFP, GFP-PRICKLE1 or GFP-PRICKLE2

PC12 neuronal-like cell lines are used as a model system for investigating neuronal differentiation in culture.[10] As reported before, a PC6-3 (sub-clone of PC12 cells) clonal line, stably expressing the tet-repressor protein pcDNA6/TR (PC6-3/TR), was generously provided by Pedro Gonzalez-Alegre (University of Iowa, Iowa City).[1] Clonal cell lines inducibly expressing GFP, GFP-PRICKLE1 or GFP-PRICKLE2 were generated as previously described.[1, 38] GFP, GFP-PRICKLE1, and GFP-PRICKLE2 cDNAs were cloned into EcoRI and XhoI restriction sites of pcDNA_5_ TO (Invitrogen). Plasmids were transfected into the PC6-3/TR cells, with Lipofectamine 2000 (Qiagen), according to the manufacturer’s instructions. Clonal cells stably expressing the transgenes were selected (medium supplemented with hygromycin at 100μg/ml) and blasticidin (5μg/ml) and screened for doxycycline inducibility by fluorescence microscopy and anti-GFP Western blots.

### Western blots

Dox-treated and untreated PC12 cells expressing GFP, GFP-PRICKLE1, or GFP-PRICKLE2 were lysed in ice-cold, NET-100 buffer (Tris 50mM, NaCl 100mM, EDTA 5mM supplemented with protease inhibitor (1X EDTA-free Complete Mini-tabs protease inhibitor cocktail from Roche). Equal amounts of proteins were resolved by sodium dodecyl sulfate-acrylamide gel electrophoresis (SDS-PAGE) in 4–20% acrylamide gels (pre-cast gels, Biorad) and transferred onto a Polyvinylidene fluoride (PVDF) membrane for 3 hours at 0.30 Amps. The membrane was blocked in 5% non-fat milk for 2 hours at room temperature followed by incubation in anti-GFP antibody at 1:1000 dilution overnight at 4°C. The membrane was then washed in TBST (Tris-buffered saline with Tween-20) at room temperature, followed by incubation in (horseradish) HRP-conjugated goat-anti-rabbit antibody (1:10 000) at room temperature for 2 hours. The blots were developed using ECL detection kit (Thermo Scientific) after washing, as per the manufacturer’s instructions. Signals were captured on x-ray films.

### Immunoprecipitation

Immunoprecipitations were carried out as previously described.[1, 38] Differentiated, dox-treated GFP, GFP-PRICKLE1, and GFP-PRICKLE2 PC12 cell lines were lysed in ice-cold NET-100 buffer supplemented with a protease inhibitor. Lysates were immunoprecipitated overnight with GFP-conjugated agarose beads after 1 hour of pre-clearing in A/G agarose beads. After 5 X 5 minute washes in NET-100 buffer, immunoprecipitates were eluted in 2X Laemmli buffer at 100°C for 5 minutes. Equal volumes of immunoprecipitates were resolved by SDG-PAGE in 4–20% acrylamide gels and then silver-stained.

### Silver staining

Following electrophoresis, a gel with resolved GFP immunoprecipitates was incubated in fixing solution (50% methanol, 10% acetic acid) for 30 minutes at room temperature and washed overnight in water. The gel was then incubated in 100mL of sodium thiosulphate solution (0.33g sodium thiosulphate/ 1L water) for 120 seconds, followed by 3 X 30-second washes in water. This was followed by incubation in silver nitrate solution (0.2g silver nitrate/100mL water) for 30 minutes. The gel was then washed in distilled water for 3 X 60 seconds, and then incubated in developing solution (3g sodium carbonate, 50μl formaldehyde, 2mL sodium thiosulphate solution, 93mL water) until proteins bands became visible. The stop solution (7g EDTA/500ML water) was added to the gel and shaken for 10 minutes to stop further development.

### Mass spectrometry LC-MS/MS

This was carried out as previously described.[1]

*SDS-PAGE*. 20 μL of clarified, soluble GFP, GFP-PRICKLE1, and GFP-PRICKLE2 immunoprecipitates were added to denaturing, SDS-PAGE, loading buffer (containing glycerin, beta-mercaptoethanol, and SDS in Tris buffer) and boiled for five minutes in preparation for electrophoresis. Bio-Rad precast 4–20% Tris-HCl gradient SDS-PAGE gels were run at 150 V for 45 minutes. Gels were then stained with Bio-Rad Flamingo fluorescent stain and imaged using a UVP PhotoDoc-It UV Imaging System (Upland, CA) followed by LC-MS/MS as described.

#### Data analysis

MS/MS data were analyzed and matched to rat protein sequences in the Swiss Prot and TrEMBL database, using the MASCOT 2.4 database search engine (Matrix Science, UK) with carbamidomethyl as a fixed modification and oxidation as a single variable modification. Mass window for the parent ions were set to 1.8 m/z and 0.4 m/z for MS/MS data. The same spectra were searched using similar restrictions with the SpectrumMill algorithm (Agilent) and alignments were merged and curated using Scaffold v3.6.4. A minimum peptide-ion score cut-off was set at 9 in SpectrumMill and the presence of at least six consecutive y- or b-ions was required. Alignments reported from Scaffold were restricted to a false discover rate of less than 1%, with peptide and protein confidence >90% and at least two unique peptides required.

#### Network analysis

We used the MetaCore (MetaCore, GeneGO Inc., St. Joseph, MI, USA) networking function and String database 9.1 to curate interaction maps of the proteins identified. Information for identified interactions is obtained from several sources including but not limited to genomic context, database imports (PPI and pathway databases), high-throughput experiments, co-expression, and text mining. We uploaded our lists of proteins from LC-MS/MS into the software programs and exported the networks into Cytoscape 2.7.0 for manipulation of the network appearance.

### Co-immunoprecipitation

HEK293T[37] cells were transfected with Flag-PRICKLE1 or Flag-PRICKLE2 with Polyfect (Qiagen), according to the manufacturer’s protocol. Cells were lysed in ice-cold NET-100 buffer after 48 hrs of incubation and immunoprecipitated overnight with anti-Flag beads at 4°C. Beads were washed for 5 minutes x 5 times in ice-cold NET-100 buffer. Immunoprecipitates were resolved by SDS-PAGE on a 4–20% gel and then subjected to anti-USP9X (1:500) immunoblot analysis. HRP-conjugated anti-rabbit secondary antibody was used at a 1: 2000 dilution. For mapping studies, HEK293T cells transfected with Flag-PRICKLE1, Flag-NPRICKLE1 or Flag-CPRICKLE1; Flag-PRICKLE2, Flag-NPRICKLE2 or Flag-CPRICKLE2 with Polyfect (Qiagen). Transfected cells were treated as described above. Lysates were also immunoprecipitated overnight with anti-Flag beads, eluates resolved by SDS-PAGE and then subjected to anti-USP9X Western blot analysis. Membranes were stripped and reprobed with anti-BCR antibodies (1:1000) overnight. HRP-conjugated anti-rabbit secondary antibody was used at a 1:10000 dilution. Blots were developed; and signals captured on X-ray films.

### Ubiquitination/deubiquitination assays

Flag-PRICKLE1 only, Flag-PRICKLE1 + HA-UbiquitinC or Flag-PRICKLE1 + HA-UbiquitinC + mCherryC1Usp9x were transfected into HEK293T cells and incubated for 48 hours. Cells were lysed and PRICKLE1 immunoprecipitated overnight via the Flag tag and resolved by SDS-PAGE. Ubiquitinated and de-ubiquitinated PRICKLE1 was detected by anti-HA (1:1000) Western blot analysis. The membrane was incubated in anti-HA-peroxidase at room temperature for 2 hours and washed for 4 X 10 minutes in TBST. Blots were developed; and signals captured on X-ray films. The process was repeated for PRICKLE2 where Flag-PRICKLE2 only, Flag-PRICKLE2 + HA-UbiquitinC or Flag-PRICKLE2 + HA-UbiquitinC + mCherryC1Usp9x were transfected. The process was repeated with full-length USP9X with and without the proteasome inhibitor LLNL (N-acetyl-L-leucyl-L-leucyl-L-norleucinal) at a concentration of 50μm overnight.

#### Web resources

http://www.molgen.ua.ac.be/SCN1AMutations/Home/Default.cfm
http://evs.gs.washington.edu/EVS/
www.genego.com
http://string-db.org

### Ethics statement

#### Mice subjects

All mouse work was done under ethical clearance from the Griffith University, and The Women’s and Children’s Health Network, and the South Australian Pathology Department Animal Ethics Committees. All protocols used were in accordance with the policy and guidelines of the National Health and Medical Research Council of Australia. Personnel and investigators who handled the mice were properly trained and qualified. Animals were treated humanely, discomfort and distress were minimized during the experiments and animals were monitored for signs of pain and distress. All euthanasia was performed using cervical dislocation.

#### Human subjects

All human DNA samples were collected with proper informed consent and with approval from an institutional review board (IRB).

#### Generation of Emx1-Cre mouse

To generate mice in which Usp9X was conditionally deleted from the dorsal telencephalon, Usp9X^loxP/loxP^ female mice were crossed with heterozygous Emx1-Cre males as described previously.^8^ Under this breeding regime, all male offspring receive a Usp9X loxP gene. Male offspring positive for Cre were used as Usp9X cKO and Cre negative male littermates were used as controls.[12]

#### Mouse genotyping

Mouse genotyping was performed on DNA extracted from tail tips and PCR was performed using RedExtract tissue PCR Kit (Sigma-Aldrich) as per the manufacturer’s instructions. Primers were designed to detect Cre-recombinase, F: CTGACCGTACACCAAAATTTGCCTG; R: GATAATCGCGAACATCTTCAGGTTC. Male embryos were identified using primers for the SRY region of the Y chromosome, F: GAGGCACAAGTTGGCCCAGCA; R: GGTTCCTGTCCCACTGCAGAAG.

#### Perfusion and Cryo-sectioning

Four-week old mice were anesthetized, perfused trans-cardially with 4% paraformaldehyde (PFA), then heads were drop-fixed in 4% PFA. Following fixation brains were cryo-protected in 15% sucrose at 4°C overnight, then in 30% sucrose at 4°C overnight, 1:1 ratio of 30% sucrose and O.C.T at 4°C overnight and frozen in Tissue-Tek O.C.T. compound (Sakura), then sectioned at 10 μm on a cryostat.

#### Immunohistochemistry

The coronal mouse brain sections were permeabilised in 1% sodium dodecyl sulphate (SDS) in 1X PBS for 4 minutes at room temperature. After washing 3 times with PBS, the samples were blocked in 2% Bovine serum albumin (BSA) in 1X PBS for 20 minutes, incubated with rabbit anti-Prickle2 antibody at 1/250 dilution in 2% BSA/PBS at 4°C overnight, then incubated with 1/200 dilution of goat anti-rabbit biotinylated antibody (Invitrogen) in 2% BSA/PBS for 1h and in 1/400 streptavidin-biotin-peroxidase complex (Invitrogen) in 2% BSA/PBS for 1h at room temperature, and mounted with Vectashield mounting medium with DAPI (Vector Laboratories). Images were obtained on Olympus FV1000 confocal microscope.

#### Fly lines

*Oregon-R* (*OR*) and outcrossed *prickle-spiny-legs* (*pk* ^*sple*^) lines are referred to in Ehaideb *et al*.[3] *fat facets* (*faf*) lines were obtained from the Bloomington *Drosophila* Stock Center at Indiana University (line numbers 25100 (w[*]; st^1^ faf^B3^/TM6B, Tb^1^), 25101 (w[*]; st^1^ faf^BX3^/TM6B, Tb^1^), 25107 (w[*]; st[1] faf[BX4]/TM6B, Tb[1])).

#### Modified bang-sensitivity behavioral assay

As previously described[3].

#### Drug treatment

Newly eclosed flies were collected and maintained on standard fly food for two days. On the third day, flies were starved for 6 hours by placing them in glass vials containing damp cotton. Three vials of each genotype were then transferred to vials containing 4 ml of standard fly food mixed with 40 μl of 100 mM Degrasyn (final concentration 1 mM; WP1130, Selleck Chemicals) and aged until the flies were 7 days old, before subjecting them to the fly-flip assay. Each vial contained ∼10 flies (5 males and 5 females) maintained at 25 degrees C.

#### “Fly flip” seizure assay

Seven-day old flies were transferred to an empty vial and the vial Flug (fly plug; Flystuff.com) was pushed downward until a gap of 2 cm (from the bottom of the vial to the Flug) was created, preventing flies from moving out of camera frame in order to keep track of individual flies. Six vials of flies of each genotype were then mechanically stimulated with a vortex mixer (Fisher Scientific Vortex Mixer; maximal setting of 10) for 5 seconds. After vortexing, flies were digitally recorded for 1 minute (from the start of the vortex); only flies that flipped on their back and remained at that position for at least one second were considered flies exhibiting muscle-jerk seizure activity. Tester was blinded to genotype for all tests. Flies were randomly sorted into placebo versus drug group.

#### Targeted resequencing of *USP9X*

We captured all *USP9X* exons and at least five base pairs of flanking sequence using molecular inversion probes, and performed next generation sequencing and data analysis as described previously.[39] Our resequencing cohort consisted of 284 males diagnosed with epileptic encephalopathy according to the International League Against Epilepsy (ILAE) classification criteria.[40] We considered only non-synonymous, frameshift or splice-site mutations that were rare (<2%) in the ESP6500 data set (see web resources) for further analysis. Segregation analysis using Sanger sequencing was performed where possible for variants that met these criteria, and paternity/maternity confirmed using the PowerPlex S5 system [Promega].

##### Characterization of USP9X variants in the exome variant server

*USP9X* variants were downloaded from the EVS website. Rare (<2%), hemizygous (male), non-synonymous variants considered damaging or possibly damaging by Polyphen were tabulated for the combined European-American and African-American populations and used for statistical analysis. In addition, one frameshift mutation not qualified by polyphen was included, for a total of four damaging protein-changing variants.

#### Protein accession numbers

Human-USP9X: Accession: NP_001034679.2 GI: 145309309, mouse-Usp9x; NP_033507.2 GI: 115511018; human-PRICKLE1 NP_001138354.1 GI: 23308518; human-PRICKLE2 NP_942559.1_GI: 38524620; *Drosophila* prickle NM_165508.2 GI: 442622668.

#### Statistics

To assess statistically enrichment for *USP9X* coding variants in the epilepsy cohort, a one-tailed chi-square test without Yates correction was performed using the number of mutant and wild-type alleles from male epilepsy patients and EVS male populations. For the EVS counts, the average number of individuals genotyped per variant in the database was used as the total genotype count in the chi-square test. Student’s *t*-tests were employed for the *Drosophila* ‘fly flip’ and modified bang sensitivity assays. Power to detect variants for both the human cohort and the fly assays was based on our previously published studies.[5]

## Supporting Information

S1 Fig Usp9x is a novel PRICKLE binding partner. **A.** Stably transfected PC12 cell lines inducibly express GFP. Doxycycline induction of GFP (Panel II) and differentiation in the presence of NGF after a 72-hr incubation period (Panel III). **B.** Anti-GFP Western blot shows PC12 cells expressing GFP, GFP-PRICKLE1 or GFP-PRICKLE2, under the control of tetracycline (doxycycline)-inducible promoters. **C.** Silver staining shows expressed transgenes and associating proteins in GFP, GFP-PRICKLE1, and GFP-PRICKLE2 immunoprecipitates from NGF-differentiated PC12 cell lines. Arrows point to GFP, GFP-PRICKLE1, and GFP-PRICKLE2 proteins.
(TIFF)

S2 Fig Mass spectrometry identifies interacting proteins (individual dots) in immunoprecipitates. Scatter plot shows both slopes of regressions are both over 1 (1.75 and 1.32 for PRICKLE1 and PRICKLE2 respectively), indicating that both GFP-PRICKLE1 and PRICKLE2 PC12 cell lines had more peptide hits than the GFP control for most proteins. The correlation coefficients for both PRICKLE1 and PRICKLE2 were less than 1 (0.85 and 0.34 respectively) indicating differences between PRICKLE proteins and the GFP control.
(TIFF)

S3 Fig PRICKLE is polyubiquitinated and degraded by the proteasome. **A,B.** Anti-Flag immunoprecipitates from HEK293T cells transiently overexpressing the indicated plasmids and increasing dosage of C1Usp9x shows increasing dosage of the C1Usp9x is associated with increasing deubiquitination of PRICKLE. Anti-Flag WB shows increasing stabilization of PRICKLE with increasing C1Usp9x-mediated deubiquitination. **C,D.** PRICKLE1 or PRICKLE2 transfected with the indicated constructs in the presence and absence of the proteasome inhibitor LNLL (50μm) shows accumulation of polyubiquitinated PRICKLE with LLNL treatment.
(TIFF)

S1 Table With peptide hits of >10, 11 proteins were found that specifically interacted with PRICKLE (PK) but not the GFP control from PC12 immunoprecipitates. Tanc and Bcr have previously been identified^6^ while Usp9x and the others listed are novel.
(DOCX)

S2 Table Numbers of rare variants found in epileptic encephalopathy cohort vs. EVS males in the combined European and African American population (p-value<0.05) indicate an association of *USP9X* with epilepsy in humans.
(DOCX)
