# Supplementary figures and images for: Seizures Are Regulated by Ubiquitin-specific Peptidase 9 X-linked (USP9X), a De-Ubiquitinase
Source: PLoS Genet. 2015 Mar 12;11(3):e1005022. doi: 10.1371/journal.pgen.1005022 (PMC4357451; doi:10.1371/journal.pgen.1005022)

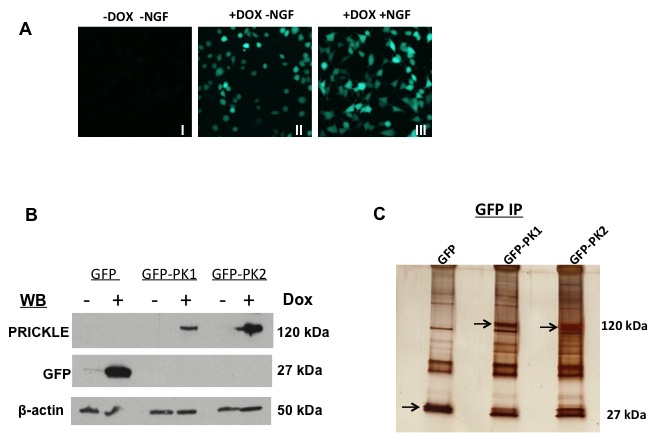

Supplement: S1 Fig — A. Stably transfected PC12 cell lines inducibly express GFP. Doxycycline induction of GFP (Panel II) and differentiation in the presence of NGF after a 72-hr incubation period (Panel III). B. Anti-GFP Western blot shows PC12 cells expressing GFP, GFP-PRICKLE1 or GFP-PRICKLE2, under the control of tetracycline (doxycycline)-inducible promoters. C. Silver staining shows expressed transgenes and associating proteins in GFP, GFP-PRICKLE1, and GFP-PRICKLE2 immunoprecipitates from NGF-differentiated PC12 cell lines. Arrows point to GFP, GFP-PRICKLE1, and GFP-PRICKLE2 proteins. (TIFF) [file pgen.1005022.s001.tiff]

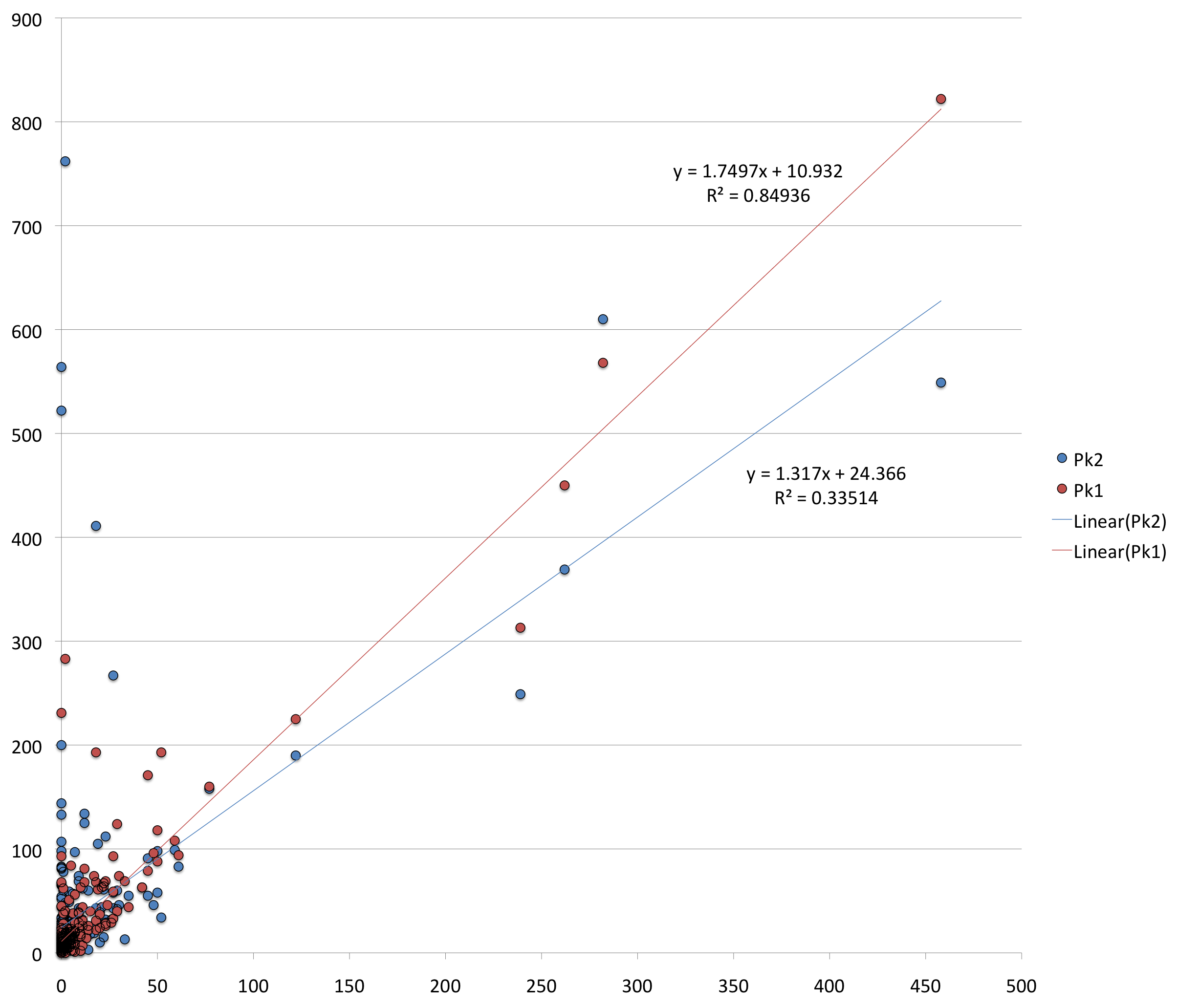

Supplement: S2 Fig — Scatter plot shows both slopes of regressions are both over 1 (1.75 and 1.32 for PRICKLE1 and PRICKLE2 respectively), indicating that both GFP-PRICKLE1 and PRICKLE2 PC12 cell lines had more peptide hits than the GFP control for most proteins. The correlation coefficients for both PRICKLE1 and PRICKLE2 were less than 1 (0.85 and 0.34 respectively) indicating differences between PRICKLE proteins and the GFP control. (TIFF) [file pgen.1005022.s002.tiff]

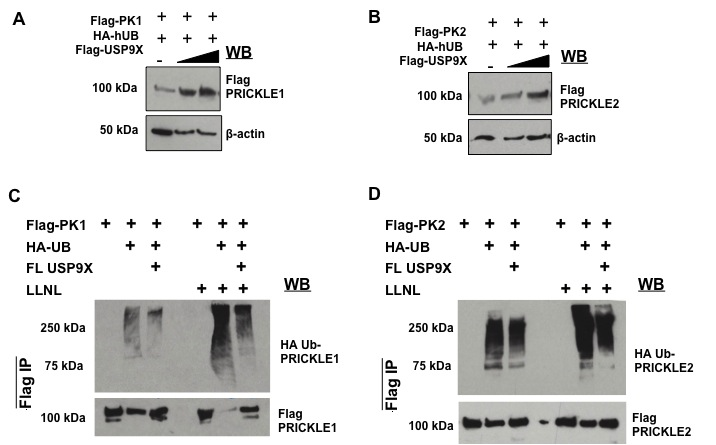

Supplement: S3 Fig — A,B. Anti-Flag immunoprecipitates from HEK293T cells transiently overexpressing the indicated plasmids and increasing dosage of C1Usp9x shows increasing dosage of the C1Usp9x is associated with increasing deubiquitination of PRICKLE. Anti-Flag WB shows increasing stabilization of PRICKLE with increasing C1Usp9x-mediated deubiquitination. C,D. PRICKLE1 or PRICKLE2 transfected with the indicated constructs in the presence and absence of the proteasome inhibitor LNLL (50μm) shows accumulation of polyubiquitinated PRICKLE with LLNL treatment. (TIFF) [file pgen.1005022.s003.tiff]
