# Supplementary material for: Seizures Are Regulated by Ubiquitin-specific Peptidase 9 X-linked (USP9X), a De-Ubiquitinase
Source: PLoS Genet. 2015 Mar 12;11(3):e1005022. doi: 10.1371/journal.pgen.1005022 (PMC4357451; doi:10.1371/journal.pgen.1005022)
Supplement: S1 Table — Tanc and Bcr have previously been identified6 while Usp9x and the others listed are novel. (DOCX) [file pgen.1005022.s004.docx]

**Supplementary Table 1**

| **Protein Name** | **Protein ID** | **GFP-PK1** | **GFP-PK2** | **GFP** |
| --- | --- | --- | --- | --- |
| Tanc2 | ENSRNOP00000052007 | 144 | 93 | 0 |
| Tanc1 | ENSRNOP00000033136 | 33 | 13 | 0 |
| Usp9x | ENSRNOP00000004814 | 522 | 68 | 0 |
| Bcr | ENSRNOP00000001766 | 81 | 45 | 0 |
| Mycbp2 | ENSRNOP00000014583 | 15 | 24 | 0 |
| Rpl23-201 | ENSRNOP00000005471 | 17 | 22 | 0 |
| Cad | ENSRNOP00000030030 | 82 | 19 | 0 |
| Rpl4 | ENSRNOP00000013462 | 66 | 16 | 0 |
| Rps26 | ENSRNOP00000007304 | 15 | 15 | 0 |
| Tubb2c | ENSRNOP00000013863 | 54 | 14 | 0 |
| Tubb5 | ENSRNOP00000001095 | 52 | 14 | 0 |
