# Supplementary material for: Seizures Are Regulated by Ubiquitin-specific Peptidase 9 X-linked (USP9X), a De-Ubiquitinase
Source: PLoS Genet. 2015 Mar 12;11(3):e1005022. doi: 10.1371/journal.pgen.1005022 (PMC4357451; doi:10.1371/journal.pgen.1005022)
Supplement: S2 Table — EVS males in the combined European and African American population (p-value<0.05) indicate an association of USP9X with epilepsy in humans. (DOCX) [file pgen.1005022.s005.docx]

**Supplementary Table 2 (Combined European American and African American populations)**

|  | VAR | WT | Total |
| --- | --- | --- | --- |
| Epilepsy males | 2 | 282 | 284 |
| EVS | 4 | 2392 | 2396 |
| Total | 6 | 2674 | 2680 |
